# Genicular artery embolization as a novel treatment for mild to moderate knee osteoarthritis: protocol design of a randomized sham-controlled clinical trial

**DOI:** 10.1186/s13063-021-05942-x

**Published:** 2022-01-08

**Authors:** T. A. van Zadelhoff, A. Moelker, S. M. A. Bierma-Zeinstra, P. K. Bos, G. P. Krestin, E. H. G. Oei

**Affiliations:** 1 Department of Radiology and Nuclear Medicine, Erasmus MC, University Medical Center Rotterdam, Rotterdam, The Netherlands; 2 Department of General Practice, Erasmus MC, University Medical Center Rotterdam, Rotterdam, The Netherlands; 3 Department Orthopaedic surgery, Erasmus MC, University Medical Center Rotterdam, Rotterdam, The Netherlands

## Abstract

**Introduction:**

Knee osteoarthritis is a common disease with pain as the most prevalent symptom. Previous cohort studies have shown genicular artery embolization to reduce pain symptoms in patients with mild to moderate knee osteoarthritis. Patients resistant to conservative therapy but not eligible yet for surgical treatment due to young age or comorbidities may profit from an effective and sustained pain reduction treatment. This study is a randomized sham-controlled trial to evaluate the efficacy of genicular artery embolization in patients with knee osteoarthritis.

**Methods and analysis:**

Fifty-eight patients with mild-to-moderate knee osteoarthritis will be recruited and randomly allocated to the treatment or control group in a 1:1 ratio. Participants in the treatment group will undergo genicular artery embolization. Patients in the control group will undergo sham treatment. Outcome measurements will be assessed at baseline and after 1, 4, 8, and 12 months with questionnaires, pressure pain threshold testing, and MR imaging. The MR imaging protocol is designed to (semi)quantitatively assess osteoarthritis in the knee joint. The primary outcome is the change from baseline of the Knee injury and Osteoarthritis Outcome Score (KOOS) pain subscale after 4 months. Secondary outcomes include change in osteoarthritis-related questionnaires, pressure pain threshold, and OA-related MRI features, particularly synovitis and bone marrow lesions.

**Ethics and dissemination:**

This trial will determine the efficacy of genicular artery embolization compared to a sham treatment. This is of importance to assess before proceeding to larger-scale efficiency studies and, ultimately, implementing this treatment into day to day clinical practice.

**Trial registration:**

ClinicalTrials.gov NCT03884049. Registered on 21 March 2019

## Introduction

Knee osteoarthritis (OA) is the most common joint disorder and a leading cause of morbidity worldwide, ranking highly on the list of contributors to disability [1]. OA also induces substantial costs both directly through medical costs as well as indirectly through loss of work productivity [2]. The most common complaint is pain. At early stages, OA is treated with exercise, weight management and topical or oral non-steroidal anti-inflammatory drugs (NSAIDs), and add on therapy with a brace and/or intra articular corticosteroid injections [3]. End-stage knee OA is treated surgically with knee joint realignment, unicompartmental or total knee arthroplasty. However, not all patients resistant to conservative therapy are automatically eligible for surgical treatment due to young age or comorbidities. Furthermore, some patients choose to not undergo surgical treatment themselves despite increasing pain and limitation [4]. There are currently no effective alternative treatments for this group of patients. It is estimated that 3.6 million Americans belong to this group and this number is projected to increase to 5 million by 2025 [5].

Recent clinical studies have shown an effective pain reduction following genicular artery embolization treatment (GAE), during which periarticular angiogenesis is embolized [6–9]. The observed improvement of pain symptoms, combined with new insights in the pathophysiology of knee OA, offer an opportunity for a novel treatment beneficial to patients in the aforementioned treatment gap. Targeting angiogenesis, which is increased in among others the synovium and the osteochondral junction, potentially reduces pain symptoms [10]. This angiogenesis is thought to be associated with synovitis, osteochondral damage and osteophyte formation. It is also accompanied by nerve ingrowth, innervating tissues that are normally not innervated. Therefore, decreasing angiogenesis through GAE may potentially have a positive effect on symptomatic knee OA.

Okuno et al. used GAE to reduce angiogenesis in 95 patients with knee OA [7]. They found a significant reduction of pain symptoms measured by the Western Ontario and McMaster Universities Osteoarthritis Index (WOMAC) pain scores (0–20 scale) from 12.1 at baseline to 2.6 after 24 months. More recently, in a study of 20 patients, Bagla et al. found a significant reduction of the mean pain visual analog score (VAS) of 76 to 29 on a 0–100 scale [8]. Although these results show promise of GAE to bridge the treatment gap for a significant proportion of knee OA patients, these previous studies were both prospective non-randomized cohort studies. Hence, it is unknown how much of the observed effect is attributable to the placebo effect. It is well known that the placebo effect plays a major role in knee OA treatment and that the type of treatment is a determinant for the magnitude of the placebo effect, which has been shown to be larger for invasive treatments [11]. To rule out the placebo effect and determine the true efficacy of GAE, a randomized controlled trial with a sham control group is mandatory. Furthermore, to investigate changes induced by GAE and understand how angiogenesis is tied in with knee OA symptoms, imaging is warranted.

Therefore, the main objective is to assess whether genicular artery embolization for patients with symptomatic knee OA results in significant pain reduction after 4 months compared to sham treatment. A secondary aim is to obtain further insight into the role of angiogenesis in knee OA pathophysiology using advanced magnetic resonance imaging (MRI). A tertiary aim is to investigate whether pain sensitization is influenced by GAE using pressure point threshold testing.

## Methods and analysis

### Study design

This study is a single-center, double blind randomized sham controlled trial. It will be performed at the Erasmus MC, University Medical Center Rotterdam, the Netherlands.

### Study population and recruitment

This is a study with multi-center recruitment from orthopedic outpatient clinics of the Erasmus MC and hospitals in the region. Written informed consent will be obtained by a clinical researcher prior to enrollment in the trial. The study population consists of patients age ≥ 18 years with symptomatic, mild-to-moderate radiographic knee OA (Kellgren and Lawrence (KL) grade 1–3) [12] resistant to conservative therapy. In order to be eligible for participation, we defined the following in- and exclusion criteria:

#### Inclusion criteria

- Age ≥ 18 years
- Knee pain for a duration of ≥ 6 months
- Knee pain (numeric rating scale ≥ 4 to ≤ 8) on at least half of the days in the preceding month at time of inclusion
- Insufficient response to conservative treatment for at least 6 months
- Radiographic knee osteoarthritis (KL grade 1–3)

#### Exclusion criteria

- Contra-indications for MRI (e.g., metallic foreign bodies, etc.)
- Contra-indications for angiography (e.g., coagulopathy)
- Previous surgical treatment for knee osteoarthritis (e.g., high tibial osteotomy), excluding knee arthroscopy
- Musculoskeletal co-morbidity (e.g., rheumatoid arthritis or gout) potentially masking the effect of GAE
- Renal insufficiency, determined with a blood sample test (GFR < 30 ml/min/1.73 m^2^)
- Known allergy to contrast agents;
- Known allergies to barium sulfate, 3-aminopropyltrialkoxysilane, polyphosphazene
- Women who are pregnant or lactating
- Intermittent claudication of affected limb
- Intra-articular injections in the ipsilateral knee less than 6 months ago
- On the waiting list for joint replacement surgery
- Amitriptyline usage
- Insufficient command of the Dutch language
- Legally incompetent adults

Participation in this trial is voluntary and patients can stop at any time without providing a reason. Patients withdrawn from the trial will be invited to fill in the questionnaires from home if they are not undergoing any other form of treatment for knee OA.

### Randomization, allocation concealment, and blinding

Patients will be randomized 1:1 using a random block size strategy with block sizes of 4 and 6. Randomization is performed using an online randomization tool (ALEA clinical: https://www.aleaclinical.eu/). The clinical researcher, blinded for randomization, will conduct the MRI scans, evaluate patients during follow-up, and will enter the patient data into an online database. The interventional radiologist who will perform the procedure will be automatically informed about the randomization result by email. The allocation will only be sent to the interventional radiologist and a third party within the department who safeguards the randomization code. All others, including the clinical researcher, treating orthopedic surgeon, and patients, will be blinded tot group allocation.

### Interventions

To ensure patients will be effectively blinded, several precautions are taken. The personnel in the intervention suite will be instructed not to mention the group the patient is allocated to or anything else that could compromise blinding status of participants. Patients will lie in supine position and wear a noise-canceling headphone with music to distract them from the procedure and surrounding sounds. The patient’s line of sight on the intervention working field and imaging monitors will be blocked by a surgical drape. The skin around the knee is cooled using ice packs to minimize non-target embolization to the skin vasculature. The groin area will be anaesthetized with 2% lidocaine 10 ml.

#### GAE procedure

Patients allocated to the treatment group will receive GAE treatment, performed by an interventional radiologist experienced in vascular embolization procedures.

Following local anesthesia, an antegrade 4 French (Fr) catheter will be inserted into the common femoral artery. Initial digital subtraction angiography targeted on the vessels around the knee will be performed using Iodixanol 320 mg I/ml (Visipaque 320, GE Healthcare, Chicago, IL, USA). Culprit vessels will be catheterized using a 1.8 Fr microcatheter with micro guidewire and embolized using Embozene Microspheres 75 μm or 100 μm (Varian Medical Systems, Palo Alto, CA, USA) dissolved in 20 ml of Visipaque 320 until stasis of flow is achieved. When no evident culprit vessel is identified, the distal branch of the genicular artery corresponding with the most painful location is embolized. After finishing the embolization procedure, the sheath will be removed, and the puncture location is manually compressed for at least 10 min after which patient is immobilized for at least 3 h, followed by discharge. The procedure time varies between 1 and 2 h.

#### Sham procedure

In the sham group, the patient setup is exactly the same as in the treatment group. After anesthesia, a small incision will be made mimicking the incision in the GAE group. The interventional radiologist will pretend to insert a catheter in the femoral artery and perform angiography and embolization. The common femoral artery will not be punctured. The C-arm of the angiography system and the table will be moved during the sham procedure mimicking the actual procedure; however, no images are actually made, so there is no unnecessary radiation exposure. The sham procedure will take approximately as long as the actual intervention.

After both procedures, the site of incision will be manually compressed for 10 min after which the patient is immobilized for at least 3 h followed by discharge. Before discharge, blinding for group allocation will be tested in all patients by asking in what group they think they were allocated to. They have 3 options: “intervention group,” “sham group,” and “I don’t know.” This timing has been chosen since testing later on in the trial could potentially be influenced by efficacy and side effects of the treatment [13].

### Co-interventions

Data on the usage of co-interventions will be collected at every follow-up visit through an interview. Patients will be encouraged not to start any new conservative treatments they were not receiving at baseline. Besides no new pharmacological treatment, this also includes no new corticosteroid injections, physiotherapy, or the usage of a brace. They can, however, increase or decrease any pharmacological treatment they were receiving based on their complaints, continue an already ongoing physiotherapy treatment, or keep wearing a brace if they were using one. This information will be recorded.

### Outcome measurements

Outcome measurements will be recorded at baseline and 1, 4, 8, and 12 months after the procedure. The primary outcome is change of the knee injury and osteoarthritis outcome score (KOOS [14]) pain subscale score between baseline and 4 months follow-up. Secondary outcomes are the change of pain subscale score between baseline and 1, 8, and 12 months. Other secondary outcomes are the change of the remaining dimensions of the KOOS (symptoms, daily living, sport and recreation and quality of life), additional questionnaires, and the pressure pain threshold after 1, 4, 8, and 12 months.

Changes on MRI will be compared between baseline and after 1 and 4 months. All clinical trial study activities are summarized in Table 1.

**Table 1:** Clinical trial study activities overview

	Study period						
	Enrolment/baseline	Intervention	Post-allocation				Close-out
Timepoint**	***− 2 weeks***	0	***Post-intervention***	***1 month***	***4 months***	***8 months***	***12 months***
**Enrolment:**							
**Eligibility screen**	**X**						
**Informed consent**	**X**						
**Randomization**	**X**						
**Allocation**	**X**						
**Interventions:**							
**Genicular artery embolization**		**X**					
**Sham procedure**		**X**					
**Assessments:**							
**Blood examination**	**X**						
**Usage of co-interventions**	**X**			**X**	**X**	**X**	**X**
**VAS pain**	**X**			**X**	**X**	**X**	**X**
**KOOS questionnaire**	**X**			**X**	**X**	**X**	**X**
**ICOAP questionnaire**	**X**			**X**	**X**	**X**	**X**
**painDETECT questionnaire**	**X**			**X**	**X**	**X**	**X**
**EQ-5D-5L questionnaire**	**X**			**X**	**X**	**X**	**X**
**Global perceived effect questionnaire**							**X**
**PPT measurement**	**X**			**X**	**X**	**X**	**X**
**MRI examination**	**X**			**X**	**X**		
**Adverse event assessment**			**X**	**X**	**X**	**X**	**X**
**Concealment of allocation assessment**			**X**				

#### Questionnaires

For the main outcome, the KOOS questionnaire is used [14]. This questionnaire consists of 42 questions divided into 5 subscales: pain (primary outcome), other symptoms, function in daily living (ADL), function in sport and recreation, and knee-related quality of life (QOL). Other questionnaires used are the Intermittent and constant osteoarthritis pain (ICOAP) [15], painDETECT [16], EuroQol 5 dimensions 5 levels (EQ-5D-5L) quality of life questionnaire [17], and a VAS (0–100 mm) score for pain, stiffness, and swelling. At the 12-month follow-up point, patients will be asked about the perceived effect of the treatment using the global perceived effect questionnaire [18]. All patients will fill in the questionnaires autonomously, but if some questions are not understood, they can ask the clinical researcher for assistance.

#### Pressure pain threshold testing

At baseline and each follow-up visit, pressure pain threshold testing (PPT) will be performed using an algometer with a tip of 1cm^2^ (Biometrics, MicroFET 2, Almere, The Netherlands). Measurements will be performed with the patient’s knee in 30° of flexion. The sites of measurements are at the medial and lateral joint space 2 cm medial and lateral to the inferior edge of patella, 2 cm above the superior border of the patella, and a peripheral site at the center of the brachioradialis muscle at the contralateral side from the treated knee. Pressure of gradually increasing intensity (10 N·s^−1^) is applied. The patient is instructed to say “stop” at the moment the sensation switches from pressure to painful. These measurements are repeated three times and are averaged to determine the pressure pain threshold. The sites are marked such that repeat measurements will be at the same location. Before measurements start, a test measurement will be done at a site not used for actual measurements for patients to get familiar with the measurements. Measurements are repeated 3 times and the average will be considered the pressure pain threshold. This method to determine the PPT has a high reliability with intra class correlation coefficients of 0.95–0.97 [19]. A cutoff limit of 120 N/cm^2^ was implemented to prevent tissue damage.

#### MRI examination

All patients will undergo MRI at baseline and at 1 and 4 months after treatment. Images will be acquired using a 3.0-T MR system with 70 cm bore (Signa Premier, GE, Chicago, IL, USA) equipped with an 18-channel dedicated knee coil (GE, Chicago, IL, USA). The MRI protocol consists of Double Echo Steady State (DESS), 3D PD (proton density) weighted fast spin echo, axial and sagittal T2 fat saturated (FS), sagittal MAGnetic resonance Image Compilation (MAGiC) synthetic imaging, dynamic contrast enhanced (DCE) MRI using DIfferential Subsampling with Cartesian Ordering (DISCO), and post contrast T1 weSPGR pulse sequences. During the DCE-MRI, 0.1 ml/kg gadovist (Bayer, Leverkusen, Germany) followed by 15 ml of saline will be administered at an injection speed of 1.0 ml/s using a contrast injector (GE, Chicago, IL, USA). This protocol was designed to assess the knee tissues semi quantitatively using the MRI Osteoarthritis Knee Score index (MOAKS [20]) and quantitatively using DCE-MRI and post contrast T1 acquisitions as well as T2-mapping of the articular cartilage using the MAGiC acquisitions.

### Adverse events and the data safety monitoring board

Adverse events (AE) are defined as any undesirable experience occurring to a subject during the study period, whether or not considered related to the intervention. One day after the procedure, the patients will be contacted and actively screened for the occurrence of any adverse events. Patients will be asked to report any changes in their health status. At every follow-up visit, patients will be actively screened for the occurrence of any AE.

Possible adverse events related to GAE are as follows: transient discoloration in the region of embolization due to non-target embolization or nerve damage due to non-target embolization. Only patients in the intervention group are subjected to these risks. All patients will receive local anesthesia and a small incision in the groin and are therefore subject to the risk of access-site hematoma and access-site infection. All patients undergo MRI with contrast agent injection and are therefore at risk for an allergic reaction to gadolinium.

After including 10 patients, a data and safety monitoring board (DSMB) will perform a safety analysis. The DSMB consists of an interventional radiologist, an orthopedic surgeon, and a biostatistician. The aim of this committee is to protect the interests of patients enrolled in this study and to evaluate the safety of the investigational intervention used in this trial. (S)AE’s and pain scores will be used to test if the intervention is deemed safe enough to continue the trial. The local ethical board will receive a report and will decide if the trial can continue. The members of this DSMB are independent of the trial. They were not involved in the creation of the protocol or have any competing interest.

Additionally, the trial conduct will be audited every 6 months by an independent auditor from the Department of Radiology & Nuclear Medicine of Erasmus MC.

### Sample size calculation

Calculations are partly based on the previous observational studies by Okuno et al. [6, 7]. Despite a possible placebo effect, which account for effect sizes up to 0.5 [21], they found effect sizes of > 5. For our study, we assumed we would require to demonstrate a large effect size of > 0.8 (strong effect) compared to sham embolization since GAE still is (minimally) invasive. Assuming this projected effect size of 0.8, and a standard deviation of 16 for the KOOS (100-0) pain score (estimated from multiple OA studies at Erasmus MC), beta of 0.80, and alpha of 0.05, 48 patients (*n* = 24 per study arm) are required to detect an increase (higher scores indicate less pain) in primary outcome measure of 8 points in the control group (placebo effect) compared to 21 points in the intervention group. The targeted sample size will be 58 to account for approximately 20% of patients lost to follow-up. We also expect a more homogeneous study population since all included subjects have an NRS pain score ≥ 4 and ≤ 8. With this sample size, and in case of lower variation in pain scores than SD of 16, we expect we could also detect smaller effect sizes between 0.5 (moderate effect) and 0.8.

### Data management

All outcomes will be entered into OpenClinica Community software (Version: 3.12.2, OpenClinica LLC and collaborators, Waltham, MA, USA, www.OpenClinica.com.), an online password protected electronic data capture system. Data will be collected anonymously. A source document review of randomly selected subjects will take place to ensure correct entering of data. All paper files will be preserved for at least 15 years.

### Statistical analysis

The primary outcome is the difference in KOOS pain subscale change from baseline to 4 months between both groups. Repeated measurements regression analysis for continuous variables will be performed, based on intention to treat analysis to determine a difference between groups. Generalized estimating equations (GEE) will be used to enable inclusion of all available measurements in the regression models even if they are correlated. It also allows us to adjust for unequal distribution of prognostic variables over the two groups, which easily can occur in smaller randomized clinical trials. Radiographic severity, pain pressure threshold, and degree of sensitized pain according to painDetect will all be tested for equal distribution and, if more than 10% different, we will test if this variable changes the estimate with more than 10%. If this is the case, the variable will be included in the adjusted analysis. The adjusted analysis will be the primary analysis, but we will also present the unadjusted analysis. If some of the patients do not receive the treatment as allocated, we will also perform a per protocol analysis where the patients that did not comply with the allocated treatment will be excluded. Repeated measurement regression analysis will also be used for all continuous secondary outcomes: KOOS subscales, VAS, ICOAP, painDETECT, EQ-5D-5L, and PPT scores.

## Discussion

GAE is a novel intervention for patients with symptomatic, mild-to-moderate knee OA [6, 7]. Initial results from cohort studies show promising outcomes with up to 4 years of pain improvement but the studies lacked a control group [5]. This randomized trial is designed to determine the efficacy of this treatment compared to a sham treatment. Sham controlled surgical trials for knee OA and degenerative meniscal tear treatment have had great impact on current insight in treatment of symptomatic knee OA. Not until sham controlled studies demonstrated no difference in pain reduction between surgery and sham surgery was the procedure removed from guidelines [21, 22]. We firmly believe that, before introducing promising new treatments, these have to be evaluated against control groups. For surgical therapies, this involves the use of sham treatment.

In our initial protocol, we planned for the sham group to undergo angiography of the genicular vessels without embolization in order to visualize and compare the angiogenesis in both groups. However, after consulting with the local ethical committee, this was deemed too invasive as a sham intervention for patients who already had knee complaints and was not necessary to achieve the primary objective. Therefore, we changed the sham treatment to be an incision in the groin without a catheter entering the femoral artery. This less invasive form of a sham procedure is a challenge with regard to blinding. Patients must be under the impression that a catheter is actually entering the arteries and that the interventional radiologist is performing the procedure. Using noise-canceling headphones so patients cannot hear their surroundings, using the C-arm to mimic angiography, and giving patients in the sham group the same post procedural care as the intervention group are examples of how we maintain adequate blinding. The upside of changing the sham procedure to this less invasive method is that patients will not be exposed to any unnecessary additional risk associated with angiography. To assess whether we succeeded to keep the intervention blinded for the patient, we will ask them for their own perception of group allocation after the treatment.

Several research groups are currently evaluating the outcome of GAE. Recently, results of non-randomized pilot studies have been published showing promising results; however, the authors also state the importance of testing GAE in a randomized controlled trial [8, 9]. Because GAE shows potential but is also invasive and incurs additional costs, a thorough evaluation is warranted before being introduced into clinical practice.

We are aware of one published protocol describing a similar trial [23]. We believe that multiple trial outcomes will strengthen the evidence upon which we can draw a conclusion on the efficacy of GAE. There are some differences to the aforementioned study protocol. Using a sophisticated MRI protocol including high-resolution DCE-MRI and testing pain pressure thresholds, we aim to expand the knowledge on the working mechanism of GAE. DCE-MRI provides quantitative measures of perfusion. This can be used to monitor changes occurring due to the alteration of angiogenesis by GAE [24]. It is suggested that DCE-MRI derived parameters are more sensitive to treatment response and associate stronger with pain changes than synovial volume [25]. If pain reduction due to GAE is truly achieved through reduction of synovitis, the perfusion parameters in the synovium should decrease commensurate with clinical improvement.

## Trial status

The current protocol version is 1.6, dated at 31 July 2020 and is approved by the local research ethics committee. As of 26 April 2020, we have included 40 patients and aim to finish inclusion in the first half of 2021. After 10 inclusions, there was a safety interim analysis by an independent data safety monitoring board. They compared adverse events occurring in both groups to confirm no unacceptable (serious) adverse events occurred in either group. The DSMB concluded the trial was safe to continue.

Furthermore, Bagla et al. noticed a reduced amount of neurogenic complications when they used 100 μm embolization particles compared to 75 μm [8]. Therefore, we added the 100 μm variant as a usable embolic agent.

## Data Availability

The datasets used and/or analyzed during the current study are available from the corresponding author on reasonable request.

## Abbreviations

OA: Osteoarthritis
NSAID: Non-steroid anti-inflammatory drugs
GAE: Genicular artery embolization
KL: Kellgren and Lawrence
Fr: French
WOMAC: Western Ontario and McMaster Universities osteoarthritis Index
VAS: Visual analog score
ICOAP: Intermittent and constant osteoarthritis pain
MRI: Magnetic resonance imaging
DCE: Dynamic contrast enhanced
MOAKS: MRI osteoarthritis knee score
KOOS: Knee injury and Osteoarthritis Outcome Score
PPT: Pressure pain threshold
MAGiC: MAGnetic resonance Image Compilation
DISCO: DIfferential Subsampling with Cartesian Ordering
weSPGR: Water excitation spoiled gradient recalled
GEE: Generalized estimating equations
DSMB: Data and safety monitoring board

## Declarations

### Communication of trial results and sharing data

Trial results will be disseminated in scientific publications. There are no plans for sharing any data.
